# Projected contraction and spatial reorganization of climatic suitability for *Glossina* in Africa under climate change

**DOI:** 10.1186/s13071-026-07599-4

**Published:** 2026-08-10

**Authors:** Ying Liu, Xiaodan Li, Huanxiang Liu, Dehua Lai, Henry H. Y. Tong

**Affiliations:** 1 Faculty of Applied Sciences, Macao Polytechnic University, Ruade Luís Gonzaga Gomes, Macau SAR, 99078 China; 2 School of Big Data, Zhuhai College of Science and Technology, Zhuhai, 519000 China; 3 Faculty of Humanities and Social Sciences, Macau Polytechnic University, Macau, China; 4 MOE Key Laboratory of Gene Function and Regulation, State Key Laboratory of Biocontrol and Guangdong Provincial Key Laboratory of Aquatic Economic Animals, School of Life Sciences, Sun Yat-Sen University, Guangzhou, 510275 China; 5 Faculty of Health Sciences and Sports, Macao Polytechnic University, Rua de Luís Gonzaga Gomes,, Macau SAR, 99078 China; 6 Centre of AI-driven Drug Discovery, Faculty of Applied Sciences, Macao Polytechnic University, Macao, China

**Keywords:** *Glossina*, Climatic suitability, Species distribution modelling, Ensemble modelling, Climate change, Africa, Vector surveillance

## Abstract

**Background:**

Tsetse flies (*Glossina* spp.), the vectors of human African trypanosomiasis, are strongly constrained by temperature and moisture conditions. Climate change may therefore alter the broad climatic envelope within which *Glossina* populations can persist across Africa.

**Methods:**

We applied an ensemble species distribution modelling framework with repeated spatial block cross-validation to estimate present-day and near-future climatic suitability for *Glossina* spp. across Africa. Occurrence records were obtained from GBIF and modelled using WorldClim bioclimatic variables. To address ecological heterogeneity within the genus, we additionally reprocessed occurrence records at the species level to assess whether species-specific or species-group models were feasible. Because no species or species group retained sufficient spatially independent records after filtering and spatial thinning, the analysis was interpreted as a continental climatic-envelope model rather than as species-specific realized distribution modelling. Projections were generated for 2021–2040 under SSP1-2.6 and SSP5-8.5.

**Results:**

Present-day climatic suitability was concentrated mainly in Central and East Africa. Temperature variability, cold-season temperature and seasonal precipitation were the most influential climatic predictors. Only GLM and RF met the predefined performance criteria and contributed to the final ensemble. Near-future projections indicated substantial contraction and spatial reorganization of climatically suitable areas under both scenarios. Using the main maxSS threshold, total suitable area declined by approximately 60% under SSP1-2.6 and 62% under SSP5-8.5. This contraction was consistent across alternative thresholds, indicating that the projected decline was not dependent on a single binary threshold. These projected changes should be interpreted within the adopted modelling framework, including the selected climate model (BCC-CSM2-MR), the retained GLM–RF ensemble and the predefined background/pseudo-absence sampling design.

**Conclusions:**

Near-future climate change is projected to substantially reduce and reorganize the broad climatic envelope for *Glossina* spp. across Africa. The estimated magnitude of contraction should be interpreted within the adopted modelling framework rather than as a universally applicable prediction. These continent-scale projections should not be interpreted as species-specific distributions or direct transmission-risk maps, but they provide a climatic baseline for climate-aware vector surveillance and future species-specific modelling.

**Graphical Abstract:**

**Figure:**
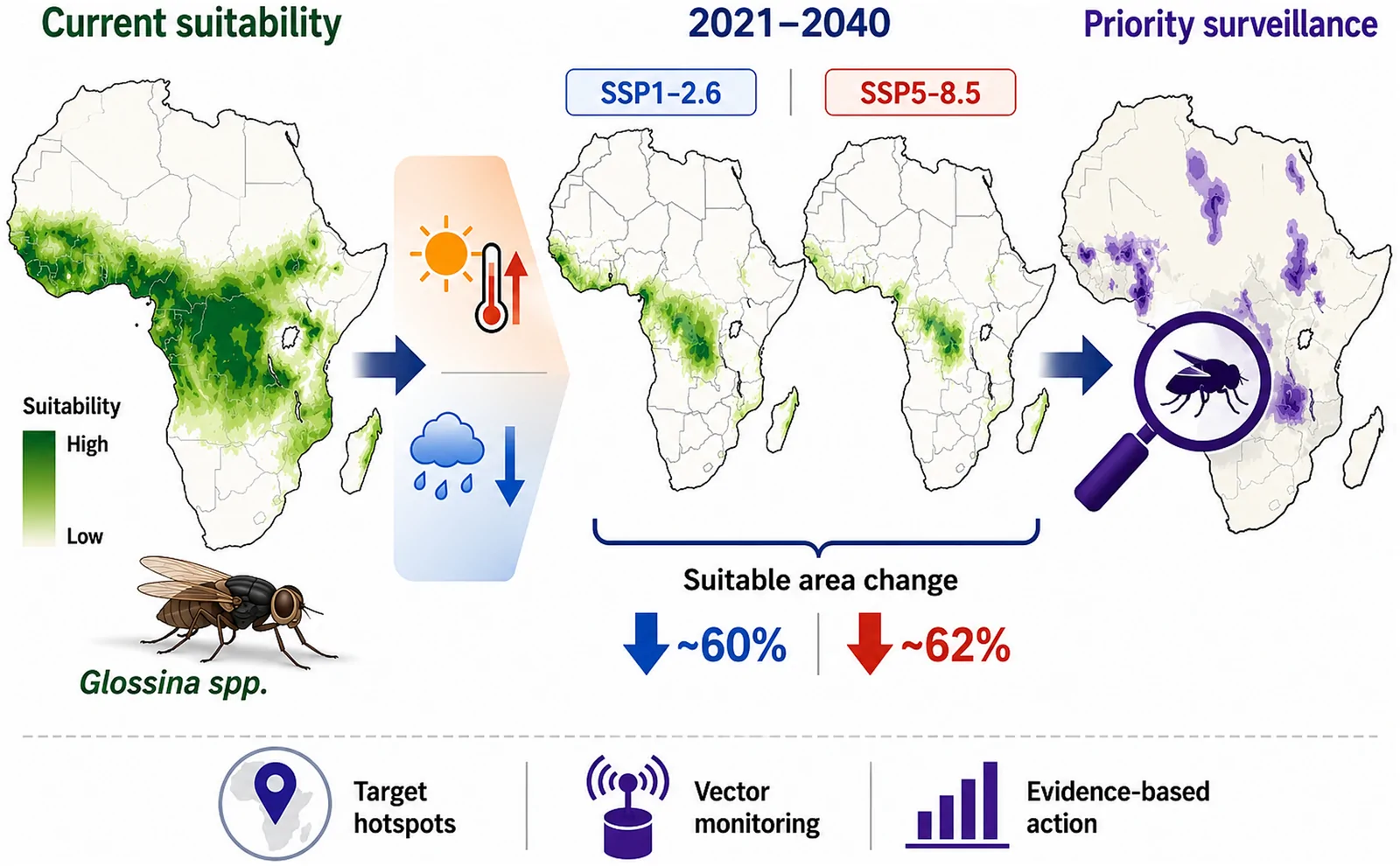

**Supplementary Information:**

The online version contains supplementary material available at https://doi.org/10.1186/s13071-026-07599-4.

## Background

Climate change is altering geographic patterns of climatic suitability for species worldwide by modifying the thermal and hydrological conditions that constrain survival, reproduction and dispersal [1–3]. Ectothermic organisms are particularly sensitive to climatic variation because their physiological performance and life-history traits are tightly coupled to ambient temperature and moisture regimes [4, 5]. Consequently, insects have exhibited rapid and spatially heterogeneous responses to recent climate change, including contractions, expansions and shifts in climatically suitable areas [6, 7].

Vector insects are of particular concern because climate-driven changes in climatic suitability may modify patterns of vector persistence and potential disease transmission [8, 9]. Among these, tsetse flies (*Glossina* spp.) are the exclusive vectors of human African trypanosomiasis (HAT) and play a central role in shaping the spatial dynamics of this disease across sub-Saharan Africa [10, 11]. The ecology of *Glossina* is strongly regulated by climatic conditions, especially temperature variability and seasonal moisture availability, which influence adult survival, larval development and long-term population persistence [12–14]. Because climate sets broad physiological constraints on tsetse survival, changes in temperature and precipitation regimes have the potential to alter the spatial configuration of climatically suitable areas across Africa.

Species distribution models (SDMs) are widely used to quantify climatic suitability and to project potential changes under future climate scenarios [15]. SDMs have been increasingly applied to vector species, including *Glossina*, to explore how climate variability and change may influence climatically suitable areas [16, 17]. However, model projections often vary among algorithms due to differences in assumptions, data requirements and sensitivity to sampling bias [18, 19]. Ensemble modelling approaches, which integrate predictions across multiple algorithms, are therefore recommended to improve robustness and to better characterize uncertainty in projected suitability patterns [18, 20].

Despite growing interest in climate-driven changes in *Glossina* suitability, several important limitations remain. Existing studies have provided valuable insights, but they differ in taxonomic focus, spatial scale and applied objective. For example, Zhou et al. [21] modelled the global potential distribution of *G. morsitans* using MaxEnt, providing a single-species assessment of climatic suitability under climate change. De Beer et al. [22] focused on *G. austeni* and *G. brevipalpis* in southern Mozambique, Eswatini and South Africa to support regional area-wide pest management. El-Hawagry et al. [23] examined *G. fuscipes* in the Afrotropical Region and Arabian Peninsula, with an emphasis on species-level distribution and future expansion. Mongare et al. [24] modelled tsetse habitat suitability in Kenya at both genus level and for *G. pallidipes*, using national atlas and field-survey data to support country-level surveillance and control planning. These studies demonstrate the value of SDMs for tsetse ecology and vector management, but they do not directly address how the broad climatic suitability landscape for *Glossina* spp. may be reorganized across the African continent in the near future. In particular, few studies have combined continent-scale modelling, spatial block validation, explicit uncertainty assessment and gain–loss analysis to evaluate persistence, contraction and potential emergence of climatically suitable areas under contrasting emission scenarios. This gap limits our ability to assess how climate change may reshape the continental climatic envelope of this important vector group.

In this study, we apply an ensemble species distribution modelling framework to quantify present-day and near-future climatic suitability for *Glossina* spp. across Africa. In response to the ecological heterogeneity among *Glossina* species, we first evaluated whether species-level or species-group modelling was feasible using the available GBIF occurrence data. Because species-specific records were insufficient after filtering and spatial thinning, our analysis explicitly focuses on the climatic component of the genus-level ecological envelope, providing a continent-scale assessment of broad climatic suitability rather than species-specific realized distributions. Specifically, we (1) evaluate the feasibility of species-level and species-group modelling; (2) assess the relative importance of climatic predictors after multicollinearity reduction; (3) map present-day climatic suitability and associated spatial uncertainty; and (4) project near-future changes in suitability under SSP1-2.6 and SSP5-8.5 for 2021–2040. By integrating spatially explicit ensemble predictions with analyses of suitability persistence, gain and loss, this study provides a climatic baseline to inform climate-aware vector surveillance and future species-specific modelling.

## Methods

### Species occurrence data

Occurrence records of African tsetse flies (*Glossina* spp.), the primary vectors of human African trypanosomiasis, used for the main genus-level SDM were obtained from the Global Biodiversity Information Facility (GBIF) [25]. These raw GBIF records were cleaned, filtered and spatially thinned before model calibration. An updated GBIF occurrence download [26] was additionally obtained for the species-level and species-group modelling-feasibility assessment. This dataset was analysed only for assessing species-level modelling feasibility and did not replace the primary genus-level occurrence dataset used throughout the SDM analysis. The relationship between the original and updated GBIF downloads and their respective analytical roles is summarized in Supplementary Table S1 and Supplementary Fig. S1.

All records were processed in R to standardize and clean geographic coordinates. Latitude and longitude fields were harmonized to decimalLatitude and decimalLongitude, converted to numeric values, and records with missing or non-finite coordinates were removed. To ensure spatial consistency with the climatic predictors, occurrences falling outside the valid WorldClim raster domain, i.e. records extracting to NA cells, were excluded.

To reduce spatial clustering and sampling bias, occurrence points were spatially filtered using a 5 arc-min grid, matching the resolution of the climatic predictors. Within each grid cell, records were randomly ordered using a fixed seed to ensure reproducibility, and up to two occurrences per cell were retained for the genus-level modelling dataset. Where available, duplicate GBIF records were removed using gbifID prior to spatial thinning. The final spatially filtered genus-level presence dataset contained 374 occurrence records and was used for model calibration, evaluation and projection. With nine climatic predictors retained in the final model, this corresponded to approximately 42 presence records per predictor. The spatial distribution of occurrence records used in this study is shown in Supplementary Fig. S2.

Because ecological differences among *Glossina* species may influence climatic suitability, we additionally evaluated whether species-level or species-group models could be fitted. For this feasibility assessment, species-level taxonomic information was retained and records were summarized after coordinate cleaning, taxonomic filtering, duplicate removal and 5 arc-min spatial thinning. Species-group feasibility was also evaluated by assigning records to the main ecological-taxonomic groups commonly used for *Glossina*: the morsitans group, the palpalis group and the fusca group.

Species-level modelling was considered feasible only when a taxon retained at least 50 spatially independent occurrence records after thinning. This operational criterion was chosen because a fivefold spatial cross-validation design would otherwise contain too few presences per fold for stable model calibration and evaluation. Species or species groups below this threshold were not modelled separately, because fitting independent SDMs with very small presence datasets would increase the risk of overfitting, unstable performance estimates and misleading projections. Species composition and modelling feasibility are reported in Supplementary Tables S2–S3. Because no individual species or species group retained sufficient spatially independent occurrence records for robust species-level modelling, the main analysis was retained as a genus-level climatic-envelope assessment for *Glossina* spp., rather than as species-specific realized distribution modelling.

### Modelling framework and candidate algorithms

We used an ensemble species distribution modelling framework to estimate climatic suitability for *Glossina* spp. across Africa. Four candidate algorithms were evaluated to represent different modelling structures: maximum entropy modelling (MaxEnt), generalized linear model (GLM), generalized additive model (GAM), and random forest (RF). MaxEnt was implemented under a presence-background framework, whereas GLM, GAM and RF were implemented using presence–pseudo-absence data. Therefore, background and pseudo-absence sampling procedures were defined before model fitting and were applied consistently across spatial cross-validation folds. Detailed algorithm settings, evaluation metrics, model-retention criteria and ensemble weighting are described in the sections below.

### Accessible area and pseudo-absence/background sampling

The accessible area for model calibration was defined at the continental scale and operationalized as the set of valid non-NA raster cells across the WorldClim climatic layers within the African modelling domain. Given the broad historical distribution of *Glossina* across sub-Saharan Africa and the continental scope of this study, the accessible area was defined broadly to reflect the macroecological domain within which climatic constraints operate. This definition constrained background and pseudo-absence sampling to climatically defined, spatially available areas and reduced unrealistic extrapolation.

Background and pseudo-absence points were generated according to the data requirements of each modelling algorithm. For MaxEnt, a presence-background design was used, contrasting presence records with background points sampled from the accessible area. For GLM, GAM and RF, pseudo-absence points were drawn from the background pool and combined with presences at a presence-to-pseudo-absence ratio of 1:5. This ratio was fixed across algorithms and cross-validation folds to maintain comparability rather than being tuned post hoc on model performance. For each spatial cross-validation fold, 10,000 background points were sampled from valid raster cells. This number was selected to characterize the broad environmental background of the African modelling domain while keeping computation feasible and consistent across folds.

To prevent information leakage, training background and pseudo-absence points were sampled only from spatial blocks assigned to the training set. Evaluation pseudo-absences were sampled from spatially independent test blocks whenever possible; when test-block sampling produced too few pseudo-absences, additional pseudo-absences were sampled from the accessible area to ensure stable metric estimation. The number of background points and the presence-to-pseudo-absence ratio were fixed across algorithms and cross-validation folds to maintain comparability; alternative background sizes or pseudo-absence ratios were not evaluated in the present analysis.

### Climatic predictors and variable selection

Climatic predictors were derived from the 19 bioclimatic variables (BIO1–BIO19), which capture annual trends, seasonality and extreme climatic conditions relevant to species distributions. Detailed definitions of these variables are provided in Supplementary Table S4. Present-day bioclimatic layers were obtained from WorldClim version 2.1 at a spatial resolution of 5 arc-min, approximately 10 km [27].

Predictor multicollinearity was reduced before model fitting using a two-step procedure conducted within the African modelling domain. First, pairwise Pearson correlations were calculated among BIO1–BIO19 using a random sample of 10,000 valid raster cells from the African climatic domain. For variable pairs with |r|> 0.8, one variable was removed, prioritizing predictors with clearer ecological interpretation for *Glossina* thermal or moisture constraints. Second, variance inflation factors (VIFs) were calculated for the retained predictor set, and only variables with VIF ≤ 10 were retained. This predictor-selection procedure was completed before model fitting and was not tuned post hoc using model outputs.

The final predictor set comprised nine variables: mean diurnal temperature range (BIO2), isothermality (BIO3), mean temperature of the wettest quarter (BIO8), mean temperature of the coldest quarter (BIO11), precipitation of the driest month (BIO14), precipitation seasonality (BIO15), precipitation of the wettest quarter (BIO16), precipitation of the warmest quarter (BIO18), and precipitation of the coldest quarter (BIO19). All retained predictors had VIF values below 10 within the African modelling domain, with values ranging from 1.78 to 6.80. No pair of retained predictors had |r|> 0.8. Details of high-correlation pairs, excluded variables, final VIF values and ecological rationale for retained variables are provided in Supplementary Table S5.

### Future climate projections

Future climate projections were derived from the same WorldClim v2.1 dataset to ensure consistency in variable definitions and spatial resolution. Bioclimatic variables for 2021–2040 under SSP1-2.6 and SSP5-8.5 were based on bias-corrected and statistically downscaled CMIP6 outputs from the Beijing Climate Center Climate System Model version 2, medium resolution (BCC-CSM2-MR), provided as pre-processed BIO1–BIO19 layers. The near-future period, 2021–2040, was selected to inform short- to medium-term vector surveillance and adaptive management planning. Mid- and late-century projections were not included because this study was deliberately focused on near-future climatic changes relevant to current surveillance and control-planning horizons.

A single GCM was used to maintain internal consistency across scenarios and to focus on algorithmic, spatial and threshold-related uncertainty within the scope of this study. Incorporating multiple GCMs would provide a broader characterization of climatic uncertainty and is addressed as a limitation in the Discussion. All present and future climatic layers were harmonized to a common spatial extent, resolution and coordinate reference system prior to projection.

### Spatial cross-validation design

To account for spatial autocorrelation and avoid inflated estimates of predictive performance, model evaluation was conducted using repeated spatial block cross-validation [28]. Presence records were assigned to spatial blocks defined on a coarse geographic grid covering the African modelling domain. Candidate block resolutions of 2.5°, 3°, 4° and 5° were evaluated using fold balance, measured as the ratio between the maximum and minimum number of presences among folds. All candidate resolutions produced the same fold-balance summary, with an imbalance ratio of 1.0135. We therefore retained the 5° block resolution for the final analysis to provide stronger spatial separation between training and testing data while maintaining balanced folds.

The final spatial partition used a 5° block resolution and consisted of five folds repeated three times. Fold allocation was highly balanced: in each repeat, the five folds contained 75, 75, 75, 75 and 74 presence records, respectively. This corresponded to an imbalance ratio of 1.0135 between the largest and smallest folds. The same spatial partitions were used consistently for model training, evaluation and permutation-based variable importance assessment. For each fold, models were trained using presence records from the training blocks, whereas evaluation was conducted using presence records from spatially independent test blocks. This design ensured spatial separation between training and testing data and reduced the influence of spatial autocorrelation on model performance estimates. Candidate block-resolution summaries and the final 5° fold balance are reported in Supplementary Table S6.

### Model calibration, evaluation and ensemble construction

All candidate models were calibrated using the same spatially filtered presence dataset and predictor subset to enable direct comparison. MaxEnt was implemented using the maxnet package under a presence-background framework, with linear, quadratic, product and hinge features and default regularization settings. GLM was fitted with a binomial error distribution and logit link. GAM was fitted with binomial smooth terms for each predictor, using s (variable, k = 4) and REML smoothing-parameter selection. RF was implemented using the ranger package as a probability forest, with 500 trees during cross-validation and 1000 trees for the final full-data prediction. Model settings are summarized in Supplementary Table S7.

For each spatial cross-validation fold, models were trained using presences and fold-specific background or pseudo-absence samples drawn from training blocks. Model evaluation was performed using presences and pseudo-absences or background samples restricted to spatially independent test blocks whenever possible. This ensured that training and evaluation data were spatially separated.

Model discrimination was assessed using the area under the receiver operating characteristic curve (AUC [29]) and the true skill statistic (TSS [30]). TSS was calculated using the threshold that maximized the sum of sensitivity and specificity [31]. Overall probabilistic prediction error was quantified using root mean square error (RMSE) between binary observations and continuous predictions. Models were retained for ensemble construction only if their mean cross-validated AUC was ≥ 0.65 and their mean cross-validated TSS was ≥ 0.25. These criteria were defined as operational retention thresholds before ensemble weighting to exclude algorithms with weak spatially independent discrimination or no positive skill. Models that did not meet the criteria were assigned zero weight.

Ensemble predictions were generated using a TSS-weighted averaging approach. For retained models, mean cross-validated TSS values were normalized to sum to one and used as ensemble weights. The final retained ensemble included RF and GLM only. RF achieved a mean AUC of 0.705 ± 0.049 and a mean TSS of 0.370 ± 0.089, receiving an ensemble weight of 0.524. GLM achieved a mean AUC of 0.668 ± 0.085 and a mean TSS of 0.337 ± 0.130, receiving an ensemble weight of 0.476. GAM did not meet the AUC retention criterion, and MaxEnt did not meet the TSS retention criterion; therefore, neither contributed to the final ensemble. Model performance, retention status and ensemble weights are reported in Supplementary Table S8.

Final suitability maps were produced by refitting the candidate models to the full dataset and predicting climatic suitability across the present-day raster stack. The ensemble mean suitability was calculated as the weighted mean of retained model predictions. Ensemble uncertainty was quantified as the weighted standard deviation among retained models. Future climatic suitability and projected changes, calculated as future minus present suitability, were generated using the corresponding future bioclimatic layers for SSP1-2.6 and SSP5-8.5.

Because the final ensemble consisted of GLM and RF, both calibrated using the same presence–pseudo-absence framework, the ensemble output was interpreted as relative climatic suitability rather than as species-specific probability of occurrence.

### Variable importance

Variable importance was estimated using permutation tests applied to spatially independent test data. Predictor values were permuted individually, predictions were recalculated and importance was quantified as the resulting decrease in AUC. If AUC could not be reliably estimated, the increase in RMSE was used instead. Raw importance values were normalized to sum to 100% within each model. Ensemble-level variable importance was calculated as the weighted average of model-specific importance values using the same weights applied in ensemble prediction.

### Binary maps, area change analysis and threshold sensitivity

To quantify changes in climatically suitable area, continuous ensemble predictions were converted to binary maps using the maxSS threshold, defined as the threshold that maximized the sum of sensitivity and specificity. The main maxSS threshold was 0.526. The same threshold was applied to present-day and future projections to ensure comparability across time periods.

Binary maps were generated for present-day conditions and future scenarios, SSP1-2.6 and SSP5-8.5, for 2021–2040. Suitable area was calculated by summing the surface area of grid cells classified as suitable using cell-specific area estimates generated with terra::cellSize. Changes between present and future conditions were classified into gain, loss, stable suitable and stable unsuitable areas. Total suitable area, absolute and percentage changes, and spatial extents of gains and losses were calculated for each scenario. Western Sahara and certain small coastal regions were excluded due to inconsistencies between land masks and climatic raster coverage in the selected WorldClim layers.

To assess whether binary suitable-area estimates were sensitive to threshold choice, we repeated the area-change analysis using three thresholds: the maxSS threshold used in the main analysis, the 10th percentile presence threshold and the equal sensitivity–specificity threshold. For each threshold, present and future suitable areas, absolute and percentage changes, and gain/loss/stable areas were recalculated under both SSP1-2.6 and SSP5-8.5. This sensitivity analysis was used to evaluate whether the direction and magnitude of projected suitable-area change were consistent across thresholding approaches. Threshold values and sensitivity results are reported in Supplementary Table S9.

### Software and reproducibility

All analyses were conducted in R (version 4.5.2; R Core Team 2025 [32]). Spatial data processing was performed using terra; data manipulation used dplyr, tidyr, readr and tibble; model fitting used maxnet, mgcv and ranger; model evaluation used pROC; and maps and figures were generated using ggplot2, patchwork and related visualization packages. Processed occurrence data, model outputs, supplementary tables and R scripts were archived with the revised submission to support reproducibility.

## Results

### Species composition and feasibility of species-level modelling

The updated GBIF download used for the species-level feasibility assessment contained 1422 raw *Glossina* occurrence records [26]. After coordinate and geographic filtering, 1421 records were retained, representing 11 identified species. Duplicate removal reduced the dataset to 98 records, and spatial thinning at 5 arc-min resolution retained 58 spatially independent records across 11 species.

No individual species retained enough spatially independent records to support a robust species-level SDM. The largest species-level datasets after thinning were *Glossina pallidipes* (*n* = 14), *G. fuscipes* (*n* = 12), *G. palpalis* (*n* = 9), *G. swynnertoni* (*n* = 8), *G. morsitans* (*n* = 5), and *G. brevipalpis* (*n* = 4). All remaining species retained only one or two spatially independent records. Species-group aggregation also remained insufficient: the morsitans group retained 27 records, the palpalis group retained 23 records, and the fusca group retained 8 records after 5 arc-min thinning.

To evaluate whether these low sample sizes were caused by the thinning resolution, we repeated the thinning at 2.5, 5 and 10 arc-min resolutions. Species-group record numbers were nearly unchanged across resolutions: the morsitans, palpalis and fusca groups retained 27, 24 and 8 records at 2.5 arc-min; 27, 23 and 8 records at 5 arc-min; and 27, 23 and 8 records at 10 arc-min, respectively. Thus, the limitation reflected the scarcity of publicly available GBIF records rather than the thinning procedure itself.

Because no individual species or species group reached the minimum data requirement for repeated spatial cross-validation and multi-algorithm modelling, separate species-level SDMs were not fitted. The main analysis was therefore retained as a genus-level climatic-envelope model, with interpretation explicitly limited to broad-scale climatic suitability for *Glossina* spp. across Africa rather than species-specific realized distributions.

### Model performance and ensemble composition

Model performance differed among algorithms under repeated spatial block cross-validation (Supplementary Table S8). RF showed the highest overall performance, with a mean AUC of 0.705 ± 0.049, mean TSS of 0.370 ± 0.089 and mean RMSE of 0.440 ± 0.022. GLM also met the predefined retention criteria, with a mean AUC of 0.668 ± 0.085, mean TSS of 0.337 ± 0.130 and mean RMSE of 0.447 ± 0.021. GAM showed moderate performance but did not meet the AUC retention threshold (AUC = 0.648 ± 0.049; TSS = 0.296 ± 0.075; RMSE = 0.451 ± 0.027). MaxEnt achieved a moderate AUC (0.700 ± 0.043), but its TSS was zero under spatially independent evaluation, indicating limited binary discrimination under the selected thresholding framework.

Based on the predefined retention criteria of mean AUC ≥ 0.65 and mean TSS ≥ 0.25, only RF and GLM were retained for ensemble construction. Their TSS-derived normalized weights were 0.524 for RF and 0.476 for GLM. GAM and MaxEnt were assigned zero weight and did not contribute to the final ensemble. The final ensemble should therefore be interpreted as a conservative GLM–RF ensemble based on the algorithms that generalized best under spatial block cross-validation.

### Climatic predictor importance

Variable-importance patterns differed between the two retained algorithms, reflecting differences between the linear structure of GLM and the non-linear tree-based structure of RF (Fig. 1). Ensemble-level variable importance therefore represents a TSS-weighted synthesis of algorithm-specific signals rather than strict convergence among all candidate algorithms.

**Figure:**
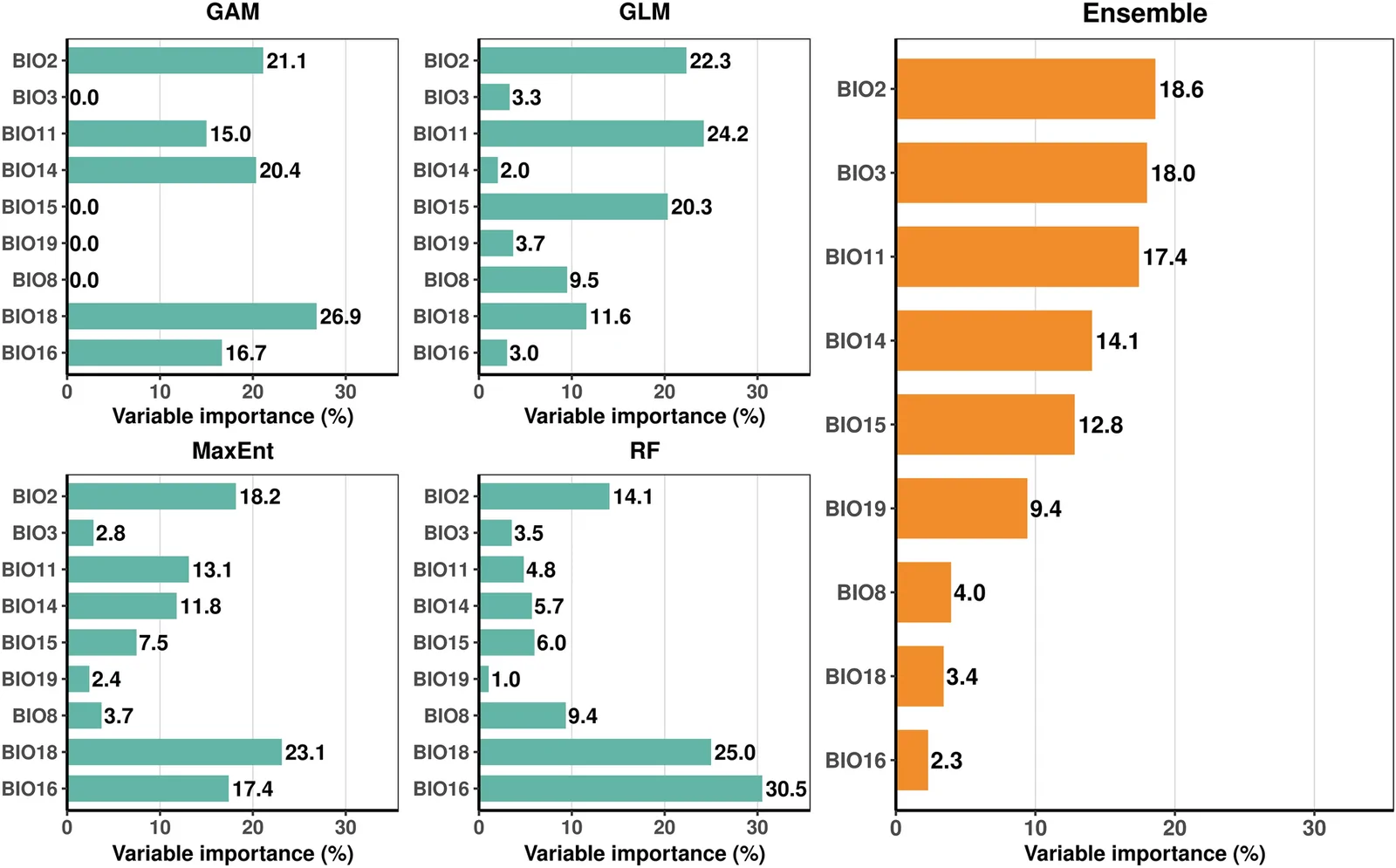
Relative importance of climatic predictors across candidate models and the final ensemble. Candidate models are shown for comparison; only GLM and RF contributed to the final ensemble

Across the retained models, predictors related to temperature variability and seasonal moisture were most influential. In the ensemble model, mean diurnal temperature range (BIO2) and isothermality (BIO3) together accounted for approximately 37% of total importance. Mean temperature of the coldest quarter (BIO11) contributed 17.4%, indicating the importance of cold-season thermal constraints. Precipitation of the driest month (BIO14) and precipitation seasonality (BIO15) together contributed 26.9%, highlighting the role of moisture limitation and seasonal precipitation variability. Overall, ensemble importance emphasized the combined influence of thermal variability, cold-season temperature and seasonal moisture availability in shaping broad climatic suitability for *Glossina* spp.

### Present-day climatic suitability patterns across Africa

The ensemble projection under present-day climate identified extensive areas of moderate to high climatic suitability for *Glossina* spp. across sub-Saharan Africa (Fig. 2A). Core areas of higher suitability were concentrated in Central Africa and extended into parts of East Africa. In contrast, northern Africa, the Sahara, much of the Horn of Africa, and southern arid and semi-arid regions consistently showed low climatic suitability.

**Figure:**
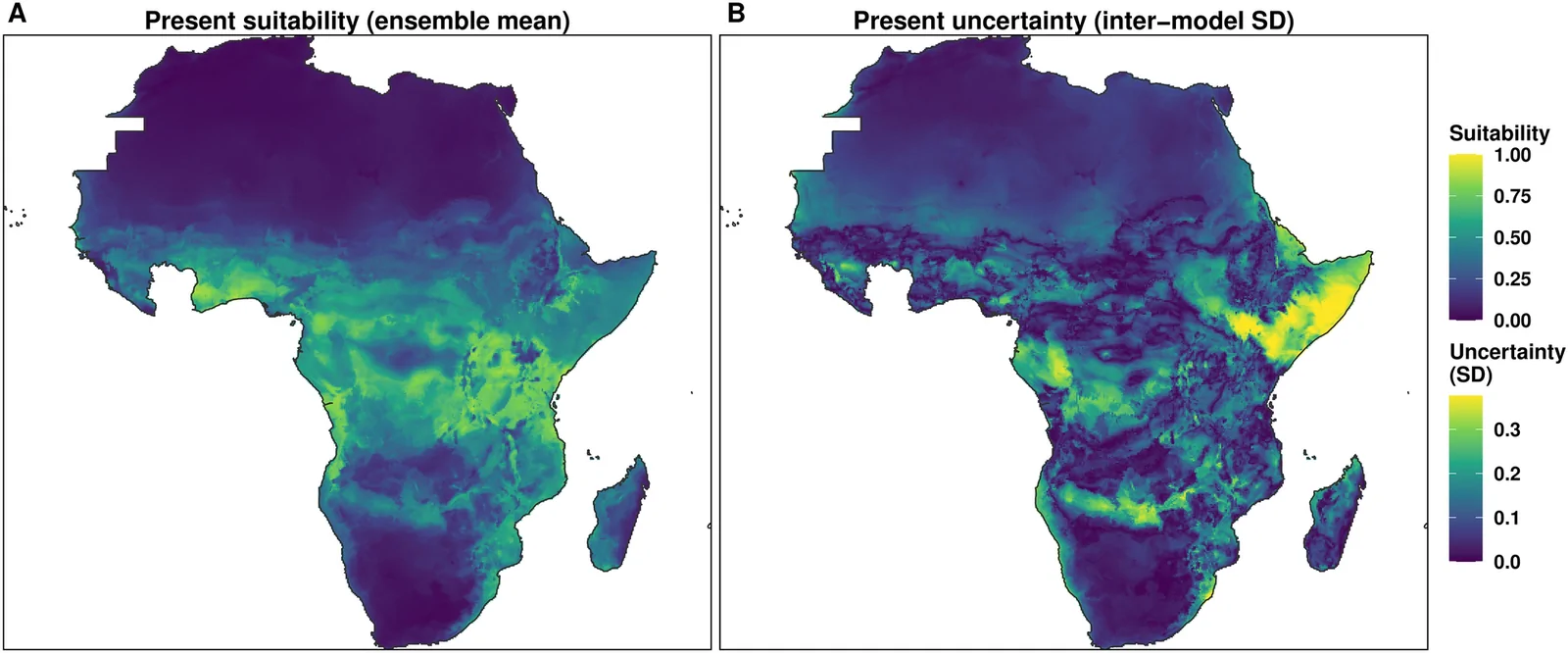
Present-day climatic suitability for *Glossina* spp. across Africa and associated inter-model uncertainty. **A** Ensemble mean climatic suitability (0–1). **B** Spatial uncertainty quantified as the inter-model standard deviation across retained modelling algorithms. African country boundaries are shown for geographic reference

Spatial uncertainty, quantified as the inter-model standard deviation across the retained GLM and RF models, was generally highest in regions of intermediate suitability and lower in areas consistently predicted as highly suitable or unsuitable (Fig. 2B). This pattern indicates that disagreement between retained models was greatest near climatic transition zones, whereas broad areas of low suitability and core suitable regions were more consistently identified.

### Projected changes in climatic suitability under future climate scenarios

Near-future ensemble projections indicated a general decline and spatial reorganization of climatic suitability for *Glossina* spp. under both climate scenarios (Fig. 3). For 2021–2040, areas of relatively higher suitability remained concentrated across equatorial and subequatorial regions of Central and East Africa under both SSP1-2.6 and SSP5-8.5 (Fig. 3A and B). However, suitability values declined across substantial portions of the present suitable range.

**F:**
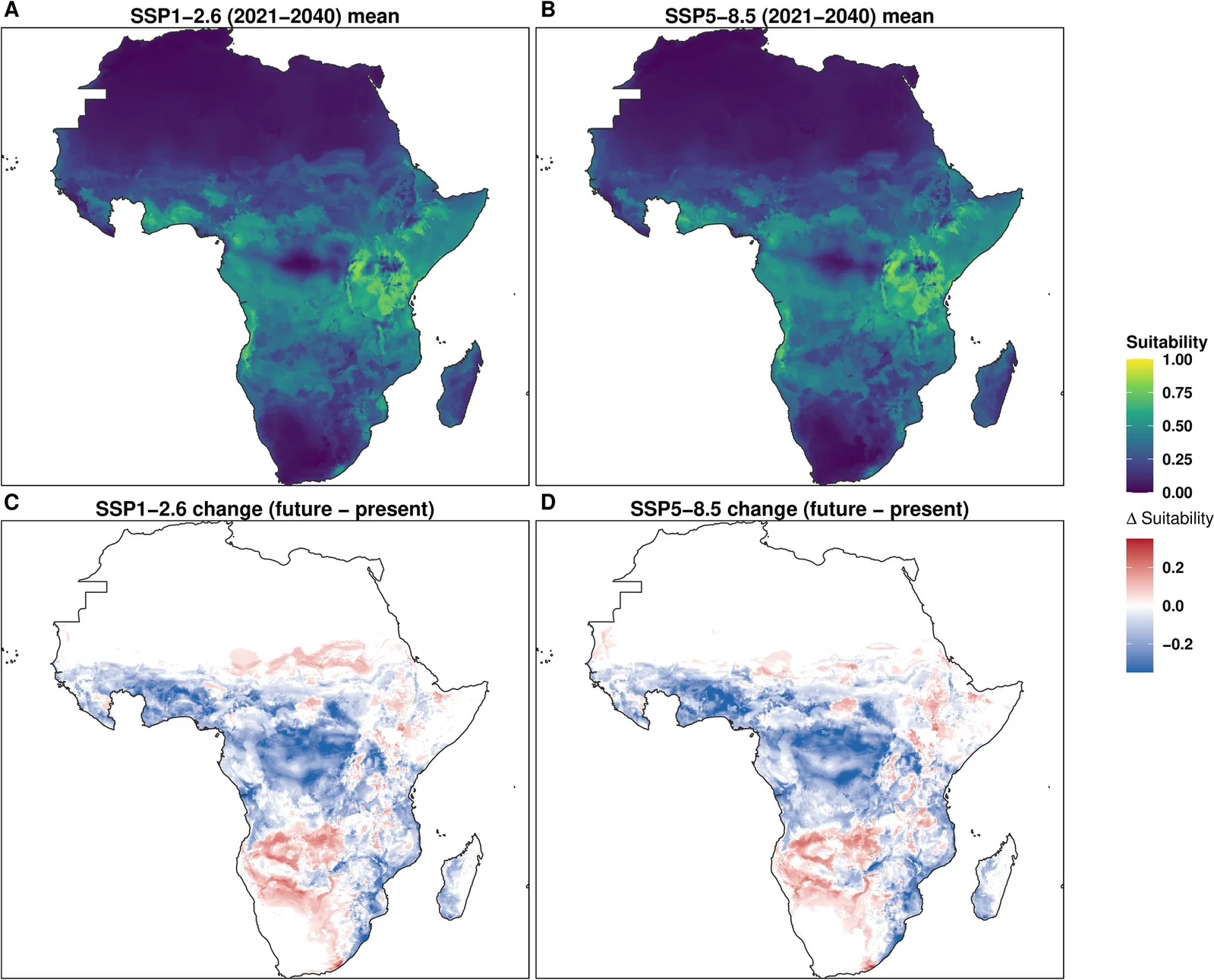
Projected changes in climatic suitability for *Glossina* spp. across Africa under future climate scenarios. **A**–**B** Ensemble mean climatic suitability for 2021–2040 under SSP1-2.6 and SSP5-8.5. **C**–**D** Changes relative to present-day conditions (future − present), with red indicating increases and blue indicating decreases in suitability

Under SSP1-2.6, changes relative to present conditions were spatially heterogeneous and moderate in magnitude (Fig. 3C). Localized gains and losses formed a mosaic pattern across parts of central, eastern and southern Africa, with declines generally more widespread than increases. Under SSP5-8.5, declines were more spatially coherent and stronger in magnitude (Fig. 3D). Reductions in suitability were particularly evident across broad areas of Central Africa, whereas areas of increasing suitability remained limited and fragmented.

Overall, the continuous suitability projections indicated broadly similar directions of change under both scenarios, with declines appearing more spatially coherent under SSP5-8.5. These patterns suggest that near-future climate change may not simply shift suitability uniformly, but instead may erode parts of the current climatic envelope while leaving a more restricted set of persistent suitable areas.

### Changes in climatically suitable area

Binary change analysis using the main maxSS threshold of 0.526 indicated substantial contraction and spatial reorganization of climatically suitable areas under both future scenarios (Fig. 4; Supplementary Table S9). Under present-day conditions, the climatically suitable area was estimated at 5.50 million km^2^. Under SSP1-2.6, suitable area declined to 2.19 million km^2^, corresponding to an approximate reduction of 3.30 million km^2^, or 60.07%. Under SSP5-8.5, suitable area declined to 2.06 million km^2^, corresponding to an approximate reduction of 3.43 million km^2^, or 62.46%.

**Figure:**
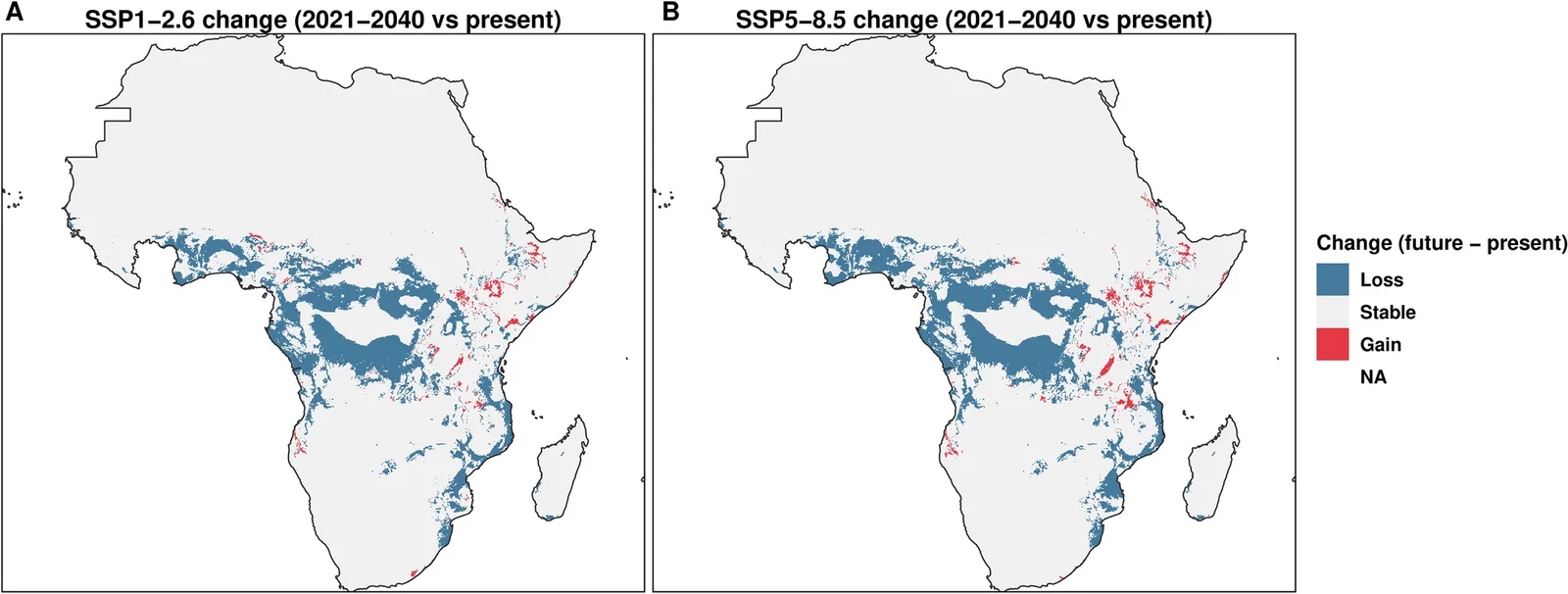
Spatial patterns of gain, loss and stability in climatic suitability for *Glossina* spp. under future climate scenarios. **A**–**B** Binary change maps showing areas of suitability loss, stability and gain between present-day conditions and projections for 2021–2040 under SSP1-2.6 and SSP5-8.5, respectively. Binary classifications were derived from ensemble climatic suitability projections using the maxSS threshold of 0.526

In the binary change maps, areas classified as suitability loss were much larger than areas classified as gain under both scenarios. Under SSP1-2.6, loss covered approximately 3.43 million km^2^, whereas gain covered only 0.19 million km^2^. Under SSP5-8.5, loss increased to approximately 3.60 million km^2^, while gain remained limited at 0.23 million km^2^. Stable suitable areas were mainly concentrated in equatorial and eastern regions, indicating partial persistence of the present climatic envelope but with substantial contraction relative to current conditions.

Spatially, suitability loss dominated across large portions of Central and southern Africa under SSP1-2.6, while gains were limited and primarily located along the margins of the present suitable range, including parts of the eastern African highlands. Under SSP5-8.5, losses became more extensive and spatially coherent, and gains remained geographically restricted and fragmented. These results indicate an asymmetric pattern of climatic suitability change, characterized by widespread loss and limited compensatory gain.

### Threshold sensitivity of suitable-area change

Threshold-sensitivity analysis showed that the projected contraction of climatically suitable area was consistent across the thresholds tested (Supplementary Table S9). Under the main maxSS threshold of 0.526, suitable area declined by 60.07% under SSP1-2.6 and 62.46% under SSP5-8.5. Using the 10th percentile presence threshold of 0.569, suitable area declined by 65.59% and 66.94%, respectively. Using the equal sensitivity–specificity threshold of 0.573, suitable area declined by 66.19% and 67.43%, respectively.

Thus, all tested thresholds indicated contraction under both future scenarios. Across the three thresholds, the projected decline ranged from 60.07% to 66.19% under SSP1-2.6 and from 62.46% to 67.43% under SSP5-8.5. This consistency suggests that the projected contraction was not an artefact of the maxSS threshold alone.

## Discussion

### Continental-scale reorganization of the *Glossina* climatic envelope

Using an ensemble species distribution modelling framework, this study provides a continent-scale assessment of present-day and near-future climatic suitability for *Glossina* spp. across Africa. Our results suggest that projected climate change may substantially reorganize the spatial configuration of climatically suitable areas over the coming decades. Under both SSP1-2.6 and SSP5-8.5, climatically suitable areas were projected to contract, with declines appearing more spatially coherent under the high-emission scenario.

The projected changes were not characterized by simple latitudinal or elevational shifts. Instead, they involved heterogeneous patterns of suitability loss, limited gain and contraction of climatically stable areas. This spatial reorganization is important because it suggests that climate change may alter the broad environmental context for tsetse persistence even within regions that are currently climatically suitable. The main signal was also consistent across thresholding approaches: all three tested thresholds indicated contraction under both scenarios, with suitable-area declines ranging from 60.07% to 66.19% under SSP1-2.6 and from 62.46% to 67.43% under SSP5-8.5. This consistency suggests that the projected contraction was not an artefact of the maxSS threshold alone.

These findings should be interpreted as changes in the continental climatic envelope of *Glossina* spp., rather than as species-specific predictions of realized distribution. The genus includes species with distinct ecological associations and climatic tolerances, and their responses to future climate change are unlikely to be identical. Nevertheless, the genus-level climatic-envelope approach provides a broad baseline for identifying where climatic conditions may become less favourable, remain suitable or potentially become more suitable at the continental scale.

### Relationship to previous studies and added value of this study

Several recent studies have advanced the modelling of *Glossina* climatic suitability and potential distributions, but they have addressed different taxonomic, spatial and applied questions. Zhou et al. [21] modelled the global potential distribution of *G. morsitans* using MaxEnt, providing valuable single-species insight into broad climatic suitability. De Beer et al. [22] focused on *G. austeni* and *G. brevipalpis* in the southern African tsetse belt, with a strong operational emphasis on regional area-wide pest management. El-Hawagry et al. [23] examined *G. fuscipes* in the Afrotropical Region and Arabian Peninsula, addressing a specific biogeographical question and projecting future suitability using multiple climate models. Mongare et al. [24] modelled tsetse habitat suitability in Kenya at both genus level and for *G. pallidipes*, using national atlas and field-survey data to support country-level surveillance.

The present study differs from these earlier works in three main ways. First, rather than focusing on a single species, a country or a subregional control zone, it evaluates the broad climatic envelope of *Glossina* spp. across the African continent. Second, it focuses on near-future change for 2021–2040, a period relevant to ongoing surveillance and control planning. Third, it explicitly quantifies spatial persistence, gain and loss of climatically suitable areas, rather than reporting only future suitable-area extent. Thus, the contribution of this study is not to replace species-specific or regional operational models, but to provide a continent-scale climatic baseline for understanding how the broad suitability landscape for tsetse flies may be reorganized under near-future climate change.

### Climatic determinants of suitability

Across the retained modelling algorithms, predictors associated with temperature variability and seasonal moisture availability emerged as the dominant determinants of climatic suitability. Mean diurnal temperature range, isothermality, cold-season temperature conditions, precipitation of the driest month and precipitation seasonality were among the most influential predictors. These results are consistent with physiological and demographic studies showing that *Glossina* survival, reproduction and population persistence are strongly constrained by thermal stress and moisture availability [33, 34].

Temperature-related predictors likely reflect multiple mechanisms. Daily and seasonal temperature variability can influence adult survival, larval development and pupal mortality, while cold-quarter temperature may define climatic limits in cooler or higher-elevation environments. Moisture-related predictors are also ecologically meaningful because dry-season conditions and precipitation seasonality influence vegetation structure, humidity and microhabitat conditions that are important for tsetse persistence. The combined importance of thermal variability and seasonal moisture therefore suggests that projected suitability loss may result from interacting heat and moisture constraints rather than from temperature change alone.

Although variable-importance values differed between GLM and RF, the ensemble results consistently emphasized these core climatic controls. This supports the interpretation that the projected suitability patterns primarily reflect broad climatic constraints rather than idiosyncrasies of a single algorithm.

### Present-day climatic suitability across Africa

Present-day ensemble projections identified extensive climatically suitable areas across sub-Saharan Africa, particularly within humid and sub-humid regions of Central and East Africa. In contrast, arid regions in northern and southern Africa were consistently classified as climatically unsuitable. This pattern is consistent with the known sensitivity of *Glossina* to moisture limitation and extreme thermal conditions, and with previous regional modelling studies showing that tsetse suitability declines in climatically marginal environments [24, 33].

The broad spatial pattern identified here is also qualitatively consistent with published occurrence-based tsetse distribution atlases, including the FAO continental atlas of African tsetse and animal trypanosomosis and the Kenya national tsetse atlas [35]. Both atlases identify Central and East Africa as major regions of documented *Glossina* occurrence, broadly corresponding to the regions of highest climatic suitability predicted by our ensemble model. Because these atlases summarize observed occurrence whereas our SDM estimates climatic suitability, complete spatial correspondence is neither expected nor required. Nevertheless, the qualitative agreement between independently compiled occurrence-based atlases and our present-day climatic suitability projections provides additional support for the realism of the predicted continental distribution pattern.

Spatial uncertainty was highest in regions of intermediate suitability, which likely represent climatic transition zones where small differences between retained algorithms can alter predicted suitability. By contrast, areas consistently predicted as highly suitable or unsuitable showed lower inter-model uncertainty. This spatial pattern suggests that the broad continental contrast between climatically favourable humid or sub-humid regions and unsuitable arid regions is relatively stable across retained algorithms, whereas predictions near suitability margins should be interpreted more cautiously.

### Future changes under contrasting emission scenarios

Future projections for 2021–2040 indicated substantial contraction and spatial reorganization of climatically suitable areas under both SSP1-2.6 and SSP5-8.5. Under SSP1-2.6, suitability losses were spatially heterogeneous, with areas of persistence remaining in parts of the present suitable range. Under SSP5-8.5, losses appeared more spatially coherent, suggesting stronger climatic constraints under higher-emission conditions.

Binary gain–loss analysis showed that suitability losses substantially exceeded gains in both scenarios. Under the main maxSS threshold, suitable area declined by approximately 60% under SSP1-2.6 and 62% under SSP5-8.5 relative to present conditions, with exact values reported in Supplementary Table S8. Gains were geographically restricted and fragmented, suggesting limited compensatory expansion under projected warming. The threshold-sensitivity analysis further showed that the direction of change remained consistent across alternative thresholding rules. Together, these results provide consistent evidence for net contraction in the broad climatic envelope of *Glossina* spp. under near-future climate change.

The somewhat greater decline under SSP5-8.5 is consistent with the expectation that higher-emission trajectories may intensify climatic constraints on tsetse suitability. However, contraction was projected under both scenarios, indicating that near-future changes in climatic suitability may occur even under lower-emission conditions. This has implications for surveillance planning because regions of climatic persistence, marginal contraction and transitional suitability may require different monitoring strategies. Accordingly, the magnitude of projected contraction should be interpreted within the adopted modelling framework, including the selected climate model, the retained GLM–RF ensemble and the predefined background/pseudo-absence sampling design, rather than as a universally applicable prediction.

### Interpretation and limitations

This study quantifies climatic suitability, representing the climatic component of the ecological niche. Realized distributions may differ because of non-climatic factors such as vegetation structure, land-use change, host availability, soil conditions relevant to pupal development, vector-control interventions, historical dispersal constraints and local microclimatic refugia. Therefore, the maps should be interpreted as broad climatic suitability patterns rather than direct maps of vector abundance or disease transmission risk.

A key limitation is that the main analysis was conducted at the genus level. *Glossina* includes species associated with different ecological settings, including savannah, riverine and forest environments, and these species may differ in their responses to temperature and moisture. We explicitly evaluated whether species-level or species-group modelling was feasible using the updated GBIF occurrence dataset. However, after coordinate filtering, duplicate removal and spatial thinning, no individual species retained sufficient spatially independent records for robust repeated spatial cross-validation. Species-group aggregation also remained data-limited. We therefore chose not to fit underpowered species-level SDMs, because doing so would likely produce unstable estimates and potentially misleading projections.

The final retained ensemble consisted of two algorithms, GLM and RF. Although four algorithms were initially evaluated, GAM and MaxEnt did not meet the predefined performance-retention criteria under spatial block cross-validation and were assigned zero weight. This reduced ensemble may underrepresent algorithmic uncertainty compared with a broader multi-algorithm ensemble. However, excluding poorly performing algorithms is preferable to allowing models with weak spatially independent performance to influence the final projection. We therefore interpret the final ensemble as a conservative GLM–RF estimate of relative climatic suitability and report the performance and weights of all candidate algorithms.

The low TSS of MaxEnt under spatial block cross-validation is noteworthy because MaxEnt has been widely used in previous *Glossina* SDM studies. In our analysis, MaxEnt achieved moderate AUC but no positive TSS under the binary thresholding framework, suggesting that its apparent discrimination did not translate into spatially independent binary classification skill. This may reflect sensitivity to spatial sampling bias, differences between presence-background and presence–pseudo-absence evaluation, or reduced transferability when spatial autocorrelation is minimized. We therefore excluded MaxEnt from the final ensemble rather than allowing a model with weak spatially independent classification performance to influence the projections.

The sampling design for background and pseudo-absence points should also be considered when interpreting the results. We used 10,000 background points and a fixed 1:5 presence-to-pseudo-absence ratio for GLM, GAM and RF to maintain consistency across spatial folds and algorithms. However, we did not conduct a full sensitivity analysis of alternative pseudo-absence ratios or background-point numbers. Because pseudo-absence sampling can influence regression-based SDMs, especially when occurrence data are spatially biased or environmentally clustered, future work should evaluate how alternative background extents, pseudo-absence ratios and sampling strategies affect projected suitability.

Another limitation is that future projections were based on a single GCM, BCC-CSM2-MR. Using multiple GCMs would provide a broader characterization of climate-model uncertainty and should be prioritized in future work, particularly because precipitation-related predictors contributed substantially to the final model and CMIP6 projections can differ across sub-Saharan Africa. Accordingly, the magnitude of projected suitable-area contraction should be interpreted as conditional upon the adopted modelling framework and may vary when alternative climate models are used.

Independent external validation against harmonized continent-wide surveillance datasets was not feasible within the reproducible GBIF-based framework adopted in this study. Nevertheless, the present-day ensemble projection is qualitatively consistent with published continental and national tsetse atlases, including the FAO continental atlas and the Kenya national atlas, which identify broadly similar regions of *Glossina* occurrence across Central and East Africa. Because atlas products summarize observed occurrence whereas SDMs estimate climatic suitability, this comparison provides evidence of broad ecological consistency rather than formal external validation.

Public database occurrence records may also contain residual sampling bias despite filtering and spatial thinning. Future work should refine these projections using curated species-specific occurrence datasets from national tsetse atlases, entomological surveys, published literature and regional monitoring programmes. Such datasets would allow separate projections for ecologically distinct *Glossina* species or species groups and would be particularly important for distinguishing climate responses among riverine, savannah and forest-associated vectors.

### Implications for vector monitoring

Although this study does not model disease transmission directly, projected changes in climatic suitability may alter the spatial context within which *Glossina* populations can persist. The contraction and fragmentation of climatically suitable areas suggest that future vector persistence may become increasingly concentrated within a reduced set of climatically favourable regions. Areas projected to remain suitable across scenarios may represent priority zones for sustained surveillance, while regions near suitability margins may warrant adaptive monitoring because they are more likely to experience gains, losses or unstable suitability.

These results should be integrated with entomological, ecological and epidemiological information before being translated into operational decisions. Climatic suitability alone is not sufficient to define vector presence or disease risk. In particular, vector-control campaigns, land-use change, host availability and local ecological conditions may either reinforce or override climate-driven suitability patterns at operational scales. However, continent-scale suitability projections can help identify broad areas where surveillance priorities may need to be maintained, intensified or reassessed under changing climatic conditions. In this sense, the study provides a climatic baseline that can complement local tsetse monitoring, control planning and future species-specific modelling efforts.

## Conclusions

This study provides a continent-scale assessment of present-day and near-future climatic suitability for *Glossina* spp. across Africa. By explicitly evaluating the feasibility of species-level and species-group modelling, we found that publicly available GBIF records were insufficient to support robust species-specific SDMs after filtering and spatial thinning. The analysis was therefore framed as a genus-level climatic-envelope assessment rather than as species-specific realized distribution modelling.

The retained GLM–RF ensemble indicated substantial contraction and spatial reorganization of climatically suitable areas under both SSP1-2.6 and SSP5-8.5 for 2021–2040. Although declines appeared slightly greater and more spatially coherent under SSP5-8.5, contraction occurred under both scenarios and remained consistent across alternative thresholding approaches. These projected changes should be interpreted within the adopted modelling framework and are therefore conditional upon the selected climate model (BCC-CSM2-MR), the retained GLM–RF ensemble and the predefined background/pseudo-absence sampling design. Climatic suitability was mainly associated with temperature variability, cold-season temperature and seasonal moisture availability, highlighting the importance of interacting thermal and hydrological constraints for *Glossina* persistence.

By combining spatial block cross-validation, transparent model-retention criteria, ensemble weighting, gain–loss analysis and threshold-sensitivity testing, this study provides a reproducible climatic baseline for assessing potential near-future changes in tsetse climatic suitability across Africa. These results should not be interpreted as direct predictions of vector abundance, species-specific distributions or disease transmission risk. Instead, they can support climate-aware vector surveillance by identifying broad regions of climatic persistence, contraction and transition. Future work should build on this baseline using curated species-specific occurrence records, multiple CMIP6 climate models and non-climatic ecological predictors to support more comprehensive assessments of future climatic uncertainty and operational risk.

## Supplementary Information

Supplementary Material 1.

Supplementary Material 2.

## Data Availability

The datasets supporting the conclusions of this article are included within the article. Occurrence records of Glossina used in this study are publicly available from the Global Biodiversity Information Facility (GBIF) at https://doi.org/10.15468/dl.4cpjht. Climatic variables were obtained from publicly available sources, including WorldClim and CMIP6 climate projections.

## References

[1] Parmesan C, Yohe G. A globally coherent fingerprint of climate change impacts across natural systems. Nature. 2003;421:37–42. 10.1038/nature01286. 12511946

[2] Walther G-R, Post E, Convey P, Menzel A, Parmesan C, Beebee TJC, et al. Ecological responses to recent climate change. Nature. 2002;416:389–95. 10.1038/416389a. 11919621

[3] Chen I-C, Hill JK, Ohlemüller R, Roy DB, Thomas CD. Rapid range shifts of species associated with high levels of climate warming. Science. 2011;333:1024–6. 10.1126/science.1206432. 21852500

[4] Deutsch CA, Tewksbury JJ, Huey RB, Sheldon KS, Ghalambor CK, Haak DC, et al. Impacts of climate warming on terrestrial ectotherms across latitude. Proc Natl Acad Sci U S A. 2008;105:6668–72. 10.1073/pnas.0709472105. PMC2373333 18458348

[5] Sunday JM, Bates AE, Dulvy NK. Thermal tolerance and the global redistribution of animals. Nat Clim Change. 2012;2:686–90. 10.1038/nclimate1539.

[6] Memmott J, Craze PG, Waser NM, Price MV. Global warming and the disruption of plant–pollinator interactions. Ecol Lett. 2007;10:710–7. 10.1111/j.1461-0248.2007.01061.x. 17594426

[7] Hallmann CA, Sorg M, Jongejans E, Siepel H, Hofland N, Schwan H, et al. More than 75 percent decline over 27 years in total flying insect biomass in protected areas. PLoS ONE. 2017;12:e0185809. 10.1371/journal.pone.0185809. PMC5646769 29045418

[8] Githeko AK, Lindsay SW, Confalonieri UE, Patz JA. Climate change and vector-borne diseases: a regional analysis. Bull World Health Organ. 2000;78:1136–47. PMC2560843 11019462

[9] Caminade C, McIntyre KM, Jones AE. Impact of recent and future climate change on vector-borne diseases. Ann N Y Acad Sci. 2014;1323:157–73. 10.1111/nyas.12527. PMC6378404 30120891

[10] Leak SG. Tsetse biology and ecology: their role in the epidemiology and control of trypanosomosis. Wallingford: CABI; 1999.

[11] Simarro PP, Diarra A, Ruiz Postigo JA, Franco JR, Jannin JG. The human African trypanosomiasis control and surveillance programme of the World Health Organization 2000–2009: the way forward. PLoS Negl Trop Dis. 2011;5:e1007. 10.1371/journal.pntd.0001007. PMC3042999 21364972

[12] Rogers DJ, Randolph SE. Mortality rates and population density of tsetse flies correlated with satellite imagery. Nature. 1991;351:739–41. 10.1038/351739a0. 2062367

[13] Hargrove JW. Tsetse population dynamics. In: Maudlin I, Holmes PH, Miles MA, editors. The trypanosomiases. Wallingford: CABI; 2004. p. 113–37.

[14] Moore S, Shrestha S, Tomlinson KW, Vuong H. Predicting the effect of climate change on African trypanosomiasis: integrating epidemiology with parasite and vector biology. J R Soc Interface. 2012;9:817–30. 10.1098/rsif.2011.0669. PMC3306657 22072451

[15] Elith J, Leathwick JR. Species distribution models: ecological explanation and prediction across space and time. Annu Rev Ecol Evol Syst. 2009;40:677–97. 10.1146/annurev.ecolsys.110308.120159.

[16] Peterson AT, Soberón J, Pearson RG, Anderson RP, Martínez‐Meyer E, Nakamura M, et al. Ecological niches and geographic distributions. Princeton: Princeton University Press; 2011.

[17] Simoonga C, Mubita C, Chisanga S, et al. Predicting the potential distribution of tsetse flies under climate change in Zambia. Parasit Vectors. 2018;11:610. 10.1186/s13071-018-3184-1.

[18] Araújo MB, New M. Ensemble forecasting of species distributions. Trends Ecol Evol. 2007;22:42–7. 10.1016/j.tree.2006.11.010. 17011070

[19] Thuiller W, Lafourcade B, Engler R, Araújo MB. BIOMOD – a platform for ensemble forecasting of species distributions. Ecography. 2009;32:369–73. 10.1111/j.1600-0587.2009.05742.x.

[20] Marmion M, Parviainen M, Luoto M, Heikkinen RK, Thuiller W. Evaluation of consensus methods in predictive species distribution modelling. Divers Distrib. 2009;15:59–69. 10.1111/j.1472-4642.2008.00552.x.

[21] Zhou R, Gao Y, Chang N, Gao T, Ma D, Li C, et al. Projecting the potential distribution of *Glossina morsitans* (Diptera: Glossinidae) under climate change using the MaxEnt model. Biology (Basel). 2021;10:1150. 10.3390/biology10111150. PMC8615152 34827144

[22] de Beer CJ, Dicko AH, Ntshangase J, Moyaba P, Taioe MO, Mulandane FC, et al. A distribution model for *Glossina brevipalpis* and *Glossina austeni* in Southern Mozambique, Eswatini and South Africa for enhanced area-wide integrated pest management approaches. PLoS Negl Trop Dis. 2021;15:e0009989. 10.1371/journal.pntd.0009989. PMC8659649 34843478

[23] El-Hawagry MSA, Al-Akeel R, Almadiy A, et al. Climate-driven distribution modeling of *Glossina fuscipes* (Diptera: Glossinidae) in the Afrotropical Region: current patterns, future projections, and implications for presence in the Arabian Peninsula. Environ Monit Assess. 2026;198:558. 10.1007/s10661-026-15355-5. 42087026

[24] Mongare R, Gachoki S, Adam E, et al. Species distribution modeling to predict tsetse fly (*Glossina* spp.) habitat suitability in Kenya. Parasit Vectors. 2025;18:378. 10.1186/s13071-025-06938-1. PMC12462162 40993789

[25] Global Biodiversity Information Facility. GBIF Occurrence Download. 2026. 10.15468/dl.h7nqra.

[26] Fick SE, Hijmans RJ. WorldClim 2: new 1-km spatial resolution climate surfaces for global land areas. Int J Climatol. 2017;37:4302–15. 10.1002/joc.5086.

[27] Roberts DR, Bahn V, Ciuti S, Boyce MS, Elith J, Guillera‐Arroita G, et al. Cross‐validation strategies for data with temporal, spatial, hierarchical, or phylogenetic structure. Ecography. 2017;40:913–29. 10.1111/ecog.02881.

[28] Fielding AH, Bell JF. A review of methods for the assessment of prediction errors in conservation presence/absence models. Environ Conserv. 1997;24:38–49. 10.1017/S0376892997000088.

[29] Allouche O, Tsoar A, Kadmon R. Assessing the accuracy of species distribution models: prevalence, kappa and the true skill statistic (TSS). J Appl Ecol. 2006;43:1223–32. 10.1111/j.1365-2664.2006.01214.x.

[30] Liu C, Berry PM, Dawson TP, Pearson RG. Selecting thresholds of occurrence in the prediction of species distributions. Ecography. 2005;28:385–93. 10.1111/j.0906-7590.2005.03957.x.

[31] R Core Team (2025) R: A language and environment for statistical computing. R Foundation for Statistical Computing, Vienna, Austria. 2025. https://www.R-project.org/

[32] Are EB, Hargrove JW. Extinction probabilities as a function of temperature for populations of tsetse (*Glossina* spp.). PLoS Negl Trop Dis. 2020;14:e0007769. 10.1371/journal.pntd.0007769. PMC7237048 32379749

[33] Kleynhans E, Terblanche JS. The evolution of water balance in *Glossina* (Diptera: Glossinidae): correlations with climate. Biol Lett. 2009;5:93–6. 10.1098/rsbl.2008.0545. PMC2657761 19004752

[34] Cecchi G, Paone M, de Gier J, Zhao W. The continental atlas of the distribution of tsetse flies in Africa. PAAT Technical and Scientific Series, No. 12. FAO, Rome. 2024. 10.4060/cd2022en

[35] Ngari NN, Gamba DO, Olet PA, Zhao W, Paone M, Cecchi G. Developing a national atlas to support the progressive control of tsetse-transmitted animal trypanosomosis in Kenya. Parasit Vectors. 2020;13:286. 10.1186/s13071-020-04156-5. PMC7275614 32503681

