# Supplementary material for: Projected contraction and spatial reorganization of climatic suitability for Glossina in Africa under climate change
Source: Parasit Vectors. 2026 Aug 10;19:490. doi: 10.1186/s13071-026-07599-4 (PMC13756922; doi:10.1186/s13071-026-07599-4)
Supplement: Supplementary file 1 [file 13071_2026_7599_MOESM1_ESM.docx]

# Supplementary Material

****Supplementary Fig. S1.** Comparison of the analytical workflows for the original and updated GBIF occurrence downloads.** The original GBIF occurrence download was used throughout the completed genus-level species distribution modelling (SDM) workflow, including occurrence cleaning, spatial thinning, model calibration, spatial block cross-validation, ensemble construction and future projection. The updated GBIF occurrence download was analysed separately to evaluate whether robust species-level or species-group SDMs were feasible after applying the same quality-control, duplicate-removal and spatial-thinning procedures while preserving species identity. The two downloads therefore served different analytical purposes rather than representing alternative versions of the same occurrence dataset.

**Note:** Coordinate and geographic filtering included the removal of records with missing or zero coordinates, invalid geographic coordinates, non-finite latitude or longitude values, and occurrences falling outside the WorldClim raster extent (i.e. extracting to **NA** cells).

### ****Supplementary Fig. S2.** Spatial distribution of** Glossina **occurrence records across Africa.** Spatial distribution of Glossina occurrence records used for model calibration across Africa. Points represent spatially filtered presence records after coordinate cleaning and thinning at a 5 arc-minute resolution. The map illustrates the geographic coverage of occurrence data used for species distribution modelling and spatial cross-validation.
