# Supplementary material for: Projected contraction and spatial reorganization of climatic suitability for Glossina in Africa under climate change
Source: Parasit Vectors. 2026 Aug 10;19:490. doi: 10.1186/s13071-026-07599-4 (PMC13756922; doi:10.1186/s13071-026-07599-4)
Supplement: Supplementary file 2 [file 13071_2026_7599_MOESM2_ESM.docx]

**Supplementary Tables**

**Supplementary Table S1. Comparison of the two GBIF occurrence downloads and their analytical roles.**

| ****Item**** | ****Original GBIF download**** | ****Updated GBIF download**** |
| --- | --- | --- |
| **GBIF DOI** | 10.15468/dl.4cpjht | 10.15468/dl.h7nqra |
| **Analytical purpose** | Development and evaluation of the primary genus-level SDM | Assessment of species-level and species-group modelling feasibility |
| **Taxonomic level** | Glossina spp. (genus level) | Individual Glossina species |
| **Raw download** | Original occurrence dataset used for the primary modelling workflow | 1,422 occurrence records |
| **After coordinate and geographic filtering** | Occurrence dataset retained for subsequent processing | 1,421 occurrence records |
| **Duplicate removal** | Applied before spatial thinning | Applied separately within each species |
| **Spatial thinning** | 5 arc-min thinning at the genus level | 5 arc-min thinning applied separately within each species |
| **Final dataset** | 374 spatially thinned genus-level occurrence records | 58 spatially independent occurrence records across 11 species |
| **Primary analytical outcome** | Genus-level ensemble SDM construction, evaluation and future projection | Assessment of whether species-level and species-group modelling was feasible |
| **Used for genus-level SDM fitting** | Yes | No |
| **Reason** | Primary occurrence dataset used throughout model calibration, evaluation, ensemble construction and future projection | Used exclusively to evaluate species-level modelling feasibility; not intended to replace the primary genus-level occurrence dataset |

*Note. The two GBIF downloads served different analytical purposes. The original download was used to construct the primary genus-level species distribution model. The updated download was analysed separately to evaluate whether robust species-level or species-group modelling was feasible after applying the same quality-control, duplicate-removal and spatial-thinning procedures. After processing, only* ***58 spatially independent occurrence records across 11 species*** *remained, and no individual species or species group retained sufficient records for repeated spatial block cross-validation. Consequently, no species-level or species-group SDMs were fitted.*

**Supplementary Table S2. Species-level GBIF record retention and feasibility of species-level SDMs.**

| **Species** | **Raw GBIF records** | **After coordinate/geographic filtering** | **After duplicate removal** | **After 5 arc-min spatial thinning** | **Number of countries** | **Countries** | **Feasibility statement** |
| --- | --- | --- | --- | --- | --- | --- | --- |
| Glossina pallidipes | 746 | 746 | 28 | 14 | 5 | KE; MZ; TZ; ZA; ZW | Insufficient for reliable species-level SDM without additional records |
| Glossina fuscipes | 249 | 249 | 15 | 12 | 7 | CD; CF; CG; ET; KE; TZ; UG | Insufficient for reliable species-level SDM without additional records |
| Glossina palpalis | 53 | 53 | 13 | 9 | 7 | BF; CI; CM; GH; GQ; LR; NG | Insufficient for SDM |
| Glossina swynnertoni | 305 | 305 | 20 | 8 | 2 | MZ; TZ | Insufficient for SDM |
| Glossina morsitans | 18 | 18 | 9 | 5 | 4 | CM; MZ; ZM; ZW | Insufficient for SDM |
| Glossina brevipalpis | 8 | 8 | 5 | 4 | 2 | MZ; ZA | Insufficient for SDM |
| Glossina tabaniformis | 27 | 27 | 3 | 2 | 2 | CF; GA | Insufficient for SDM |
| Glossina caliginea | 2 | 2 | 2 | 1 | 1 | GQ | Insufficient for SDM |
| Glossina medicorum | 3 | 3 | 1 | 1 | 1 | BF | Insufficient for SDM |
| Glossina pallicera | 5 | 5 | 1 | 1 | 1 | LR | Insufficient for SDM |
| Glossina tachinoides | 5 | 5 | 1 | 1 | 1 | CM | Insufficient for SDM |
| Glossina austeni | 1 | 0 | 0 | 0 |  |  | Excluded after cleaning |

*Note. Species-level modelling was considered feasible only when a species retained at least 50 spatially independent occurrence records after 5 arc-min thinning. No individual species met this criterion.*

**Supplementary Table S3a. Species-group feasibility after 5 arc-min spatial thinning.**

| **Species group** | **Records after 5 arc-min spatial thinning** | **Number of species** | **Species included** | **Number of countries** | **Countries** | **Feasibility statement** |
| --- | --- | --- | --- | --- | --- | --- |
| morsitans group | 27 | 3 | Glossina morsitans; Glossina pallidipes; Glossina swynnertoni | 7 | CM; KE; MZ; TZ; ZA; ZM; ZW | Insufficient for reliable species-group SDM without additional records |
| palpalis group | 23 | 4 | Glossina fuscipes; Glossina pallicera; Glossina palpalis; Glossina tachinoides | 14 | BF; CD; CF; CG; CI; CM; ET; GH; GQ; KE; LR; NG; TZ; UG | Insufficient for reliable species-group SDM without additional records |
| fusca group | 8 | 4 | Glossina brevipalpis; Glossina caliginea; Glossina medicorum; Glossina tabaniformis | 6 | BF; CF; GA; GQ; MZ; ZA | Insufficient for reliable species-group SDM without additional records |

*Note. Species were assigned to the morsitans, palpalis and fusca groups. No species group retained enough spatially independent records for robust species-group SDM under repeated spatial cross-validation.*

**Supplementary Table S3b. Species-group thinning sensitivity across 2.5, 5 and 10 arc-min resolutions.**

| **Thinning resolution (arc-min)** | **Species group** | **Records after thinning** | **Number of species** | **Species included** |
| --- | --- | --- | --- | --- |
| 2.5 | morsitans group | 27 | 3 | Glossina morsitans; Glossina pallidipes; Glossina swynnertoni |
| 2.5 | palpalis group | 24 | 4 | Glossina fuscipes; Glossina pallicera; Glossina palpalis; Glossina tachinoides |
| 2.5 | fusca group | 8 | 4 | Glossina brevipalpis; Glossina caliginea; Glossina medicorum; Glossina tabaniformis |
| 5.0 | morsitans group | 27 | 3 | Glossina morsitans; Glossina pallidipes; Glossina swynnertoni |
| 5.0 | palpalis group | 23 | 4 | Glossina fuscipes; Glossina pallicera; Glossina palpalis; Glossina tachinoides |
| 5.0 | fusca group | 8 | 4 | Glossina brevipalpis; Glossina caliginea; Glossina medicorum; Glossina tabaniformis |
| 10.0 | morsitans group | 27 | 3 | Glossina morsitans; Glossina pallidipes; Glossina swynnertoni |
| 10.0 | palpalis group | 23 | 4 | Glossina fuscipes; Glossina pallicera; Glossina palpalis; Glossina tachinoides |
| 10.0 | fusca group | 8 | 4 | Glossina brevipalpis; Glossina caliginea; Glossina medicorum; Glossina tabaniformis |

*Note. Record numbers changed little across thinning resolutions, indicating that species-group data limitation was not primarily caused by the selected 5 arc-min thinning resolution.*

**Supplementary Table S4. Definitions of WorldClim bioclimatic variables BIO1-BIO19.**

| **Variable** | **Definition** |
| --- | --- |
| BIO1 | Annual mean temperature |
| BIO2 | Mean diurnal range (mean of monthly maximum temperature minus minimum temperature) |
| BIO3 | Isothermality (BIO2/BIO7 x 100) |
| BIO4 | Temperature seasonality (standard deviation x 100) |
| BIO5 | Maximum temperature of the warmest month |
| BIO6 | Minimum temperature of the coldest month |
| BIO7 | Temperature annual range (BIO5-BIO6) |
| BIO8 | Mean temperature of the wettest quarter |
| BIO9 | Mean temperature of the driest quarter |
| BIO10 | Mean temperature of the warmest quarter |
| BIO11 | Mean temperature of the coldest quarter |
| BIO12 | Annual precipitation |
| BIO13 | Precipitation of the wettest month |
| BIO14 | Precipitation of the driest month |
| BIO15 | Precipitation seasonality (coefficient of variation) |
| BIO16 | Precipitation of the wettest quarter |
| BIO17 | Precipitation of the driest quarter |
| BIO18 | Precipitation of the warmest quarter |
| BIO19 | Precipitation of the coldest quarter |

*Note. Definitions follow the standard WorldClim bioclimatic variable naming convention. Temperature variables are based on degrees Celsius and precipitation variables are based on millimetres in the source data.*

**Supplementary Table S5a. Predictor selection and multicollinearity assessment for BIO1-BIO19.**

| **Variable** | **Bioclimatic variable** | **Selection status** | **Selection reason** | **Maximum absolute correlation with retained predictors** | **Final-set VIF** | **VIF pass** |
| --- | --- | --- | --- | --- | --- | --- |
| bio1 | Annual Mean Temperature | Excluded | Excluded during pairwise correlation screening because it was highly correlated with retained predictor(s): bio11 (r=0.841); bio8 (r=0.805) | 0.841 |  |  |
| bio2 | Mean Diurnal Range | Retained | Retained in the final predictor set; VIF within acceptable range in the African modelling domain. |  | 1.777 | True |
| bio3 | Isothermality | Retained | Retained in the final predictor set; VIF within acceptable range in the African modelling domain. |  | 4.373 | True |
| bio4 | Temperature Seasonality | Excluded | Excluded during pairwise correlation screening because it was highly correlated with retained predictor(s): bio3 (r=-0.93) | 0.930 |  |  |
| bio5 | Max Temperature of Warmest Month | Excluded | Excluded during multicollinearity reduction to minimize redundancy and retain an interpretable predictor set. |  |  |  |
| bio6 | Min Temperature of Coldest Month | Excluded | Excluded during pairwise correlation screening because it was highly correlated with retained predictor(s): bio11 (r=0.959) | 0.959 |  |  |
| bio7 | Temperature Annual Range | Excluded | Excluded during pairwise correlation screening because it was highly correlated with retained predictor(s): bio3 (r=-0.872) | 0.872 |  |  |
| bio8 | Mean Temperature of Wettest Quarter | Retained | Retained in the final predictor set; VIF within acceptable range in the African modelling domain. |  | 3.921 | True |
| bio9 | Mean Temperature of Driest Quarter | Excluded | Excluded during multicollinearity reduction to minimize redundancy and retain an interpretable predictor set. |  |  |  |
| bio10 | Mean Temperature of Warmest Quarter | Excluded | Excluded during multicollinearity reduction to minimize redundancy and retain an interpretable predictor set. |  |  |  |
| bio11 | Mean Temperature of Coldest Quarter | Retained | Retained in the final predictor set; VIF within acceptable range in the African modelling domain. |  | 6.795 | True |
| bio12 | Annual Precipitation | Excluded | Excluded during pairwise correlation screening because it was highly correlated with retained predictor(s): bio16 (r=0.943); bio18 (r=0.818) | 0.943 |  |  |
| bio13 | Precipitation of Wettest Month | Excluded | Excluded during pairwise correlation screening because it was highly correlated with retained predictor(s): bio16 (r=0.992) | 0.992 |  |  |
| bio14 | Precipitation of Driest Month | Retained | Retained in the final predictor set; VIF within acceptable range in the African modelling domain. |  | 2.116 | True |
| bio15 | Precipitation Seasonality | Retained | Retained in the final predictor set; VIF within acceptable range in the African modelling domain. |  | 1.794 | True |
| bio16 | Precipitation of Wettest Quarter | Retained | Retained in the final predictor set; VIF within acceptable range in the African modelling domain. |  | 4.694 | True |
| bio17 | Precipitation of Driest Quarter | Excluded | Excluded during pairwise correlation screening because it was highly correlated with retained predictor(s): bio14 (r=0.985) | 0.985 |  |  |
| bio18 | Precipitation of Warmest Quarter | Retained | Retained in the final predictor set; VIF within acceptable range in the African modelling domain. |  | 4.673 | True |
| bio19 | Precipitation of Coldest Quarter | Retained | Retained in the final predictor set; VIF within acceptable range in the African modelling domain. |  | 2.126 | True |

*Note. Pairwise Pearson correlations were calculated using 10,000 randomly sampled valid raster cells within the African modelling domain. Variables were screened using |r| > 0.8 followed by VIF assessment. Predictor selection was completed before model fitting and was not tuned post hoc using model outputs.*

**Supplementary Table S5b. Variance inflation factors for the final retained predictor set.**

| **Variable** | **VIF** | **VIF threshold** | **Pass** |
| --- | --- | --- | --- |
| bio11 | 6.795 | 10 | True |
| bio16 | 4.694 | 10 | True |
| bio18 | 4.673 | 10 | True |
| bio3 | 4.373 | 10 | True |
| bio8 | 3.921 | 10 | True |
| bio19 | 2.126 | 10 | True |
| bio14 | 2.116 | 10 | True |
| bio15 | 1.794 | 10 | True |
| bio2 | 1.777 | 10 | True |

*Note. All retained predictors had VIF values below the threshold of 10.*

**Supplementary Table S5c. High-correlation pairs among final retained predictors.**

**Result**

No retained predictor pair exceeded |r| > 0.8.

*Note. No pair of retained predictors exceeded |r| > 0.8.*

**Supplementary Table S6a. Candidate spatial block resolutions evaluated for spatial block cross-validation.**

| **Candidate block resolution (deg)** | **Number of repeats** | **Number of folds** | **Mean minimum presences per fold** | **Mean maximum presences per fold** | **Mean presences per fold** | **Mean imbalance ratio** | **Minimum imbalance ratio** | **Maximum imbalance ratio** | **Final use** |
| --- | --- | --- | --- | --- | --- | --- | --- | --- | --- |
| 2.5 | 3 | 5 | 74 | 75 | 74.8 | 1.0135 | 1.0135 | 1.0135 | Evaluated |
| 3.0 | 3 | 5 | 74 | 75 | 74.8 | 1.0135 | 1.0135 | 1.0135 | Evaluated |
| 4.0 | 3 | 5 | 74 | 75 | 74.8 | 1.0135 | 1.0135 | 1.0135 | Evaluated |
| 5.0 | 3 | 5 | 74 | 75 | 74.8 | 1.0135 | 1.0135 | 1.0135 | Selected for final analysis |

*Note. Candidate block resolutions were evaluated using fold balance, measured as the ratio between the maximum and minimum number of presences among folds. All candidate resolutions produced identical fold-balance summaries; therefore, the 5 degree block resolution was retained for the final analysis to provide stronger spatial separation between training and testing data while maintaining balanced folds.*

**Supplementary Table S6b. Fold balance under the final 5 degree spatial block cross-validation design.**

| **Repeat ID** | **Block resolution (deg)** | **Number of folds** | **Minimum presences** | **Maximum presences** | **Mean presences** | **Imbalance ratio** |
| --- | --- | --- | --- | --- | --- | --- |
| 1 | 5.0 | 5 | 74 | 75 | 74.8 | 1.0135 |
| 2 | 5.0 | 5 | 74 | 75 | 74.8 | 1.0135 |
| 3 | 5.0 | 5 | 74 | 75 | 74.8 | 1.0135 |

*Note. Each repeat consisted of five folds. The imbalance ratio was calculated as the maximum number of presences divided by the minimum number of presences among folds.*

**Supplementary Table S7. Model settings and software parameters.**

| **Algorithm** | **Modelling design** | **R package** | **Main settings** |
| --- | --- | --- | --- |
| MaxEnt | Presence-background | maxnet | maxnet.formula with feature classes = lqph |
| GLM | Presence-pseudo-absence | stats | binomial GLM with logit link |
| GAM | Presence-pseudo-absence | mgcv | binomial GAM with smooth terms s(variable, k = 4), method = REML |
| RF | Presence-pseudo-absence | ranger | probability forest; num.trees = 500 during cross-validation and 1000 for final full-data prediction; min.node.size = 5 |

*Note. All models used the same final predictor set. MaxEnt used a presence-background design, while GLM, GAM and RF used a presence-pseudo-absence design with a 1:5 presence-to-pseudo-absence ratio.*

**Supplementary Table S8. Model performance, retention status and ensemble weights under repeated spatial block cross-validation.**

| **Model** | **Ensemble weight** | **AUC mean** | **AUC SD** | **TSS mean** | **TSS SD** | **RMSE mean** | **RMSE SD** | **Passed retention criteria** |
| --- | --- | --- | --- | --- | --- | --- | --- | --- |
| RF | 0.5237 | 0.705 | 0.049 | 0.370 | 0.089 | 0.440 | 0.022 | True |
| GLM | 0.4763 | 0.668 | 0.085 | 0.337 | 0.130 | 0.447 | 0.021 | True |
| GAM | 0.0000 | 0.648 | 0.049 | 0.296 | 0.075 | 0.451 | 0.027 | False |
| MaxEnt | 0.0000 | 0.700 | 0.043 | 0.000 | 0.000 | 0.527 | 0.036 | False |

*Note. Models were retained for ensemble construction if mean AUC >= 0.65 and mean TSS >= 0.25. Ensemble weights were calculated by normalizing mean TSS values of retained models to sum to one. Models not meeting the retention criteria were assigned zero weight.*

**Supplementary Table S9a. Threshold values used for threshold-sensitivity analysis.**

| **Threshold method** | **Threshold value** |
| --- | --- |
| maxSS | 0.526174 |
| 10th_percentile_presence | 0.568544 |
| equal_sensitivity_specificity | 0.572871 |

*Note. The maxSS threshold maximized the sum of sensitivity and specificity. Alternative thresholds were used to assess whether suitable-area changes were sensitive to threshold choice.*

**Supplementary Table S9b. Threshold sensitivity of projected climatically suitable area.**

| **Threshold method** | **Threshold value** | **Scenario** | **Suitable area (km2)** | **Absolute change (km2)** | **Percent change** |
| --- | --- | --- | --- | --- | --- |
| maxSS | 0.526174 | Present | 5,495,083 |  |  |
| maxSS | 0.526174 | SSP1-2.6_2021-2040 | 2,194,257 | -3,300,826 | -60.07 |
| maxSS | 0.526174 | SSP5-8.5_2021-2040 | 2,063,024 | -3,432,059 | -62.46 |
| 10th_percentile_presence | 0.568544 | Present | 4,245,535 |  |  |
| 10th_percentile_presence | 0.568544 | SSP1-2.6_2021-2040 | 1,460,805 | -2,784,731 | -65.59 |
| 10th_percentile_presence | 0.568544 | SSP5-8.5_2021-2040 | 1,403,601 | -2,841,934 | -66.94 |
| equal_sensitivity_specificity | 0.572871 | Present | 4,137,265 |  |  |
| equal_sensitivity_specificity | 0.572871 | SSP1-2.6_2021-2040 | 1,399,015 | -2,738,250 | -66.19 |
| equal_sensitivity_specificity | 0.572871 | SSP5-8.5_2021-2040 | 1,347,587 | -2,789,678 | -67.43 |

*Note. Suitable area, absolute change and percentage change were recalculated under each threshold. Present-day area is reported separately for each threshold because binary suitable area varies with threshold choice.*

**Supplementary Table S9c. Gain, loss and stable areas under alternative thresholds.**

| **Threshold method** | **Threshold value** | **Scenario** | **Gain (km2)** | **Loss (km2)** | **Stable suitable (km2)** | **Stable unsuitable (km2)** |
| --- | --- | --- | --- | --- | --- | --- |
| maxSS | 0.526174 | SSP1-2.6_2021-2040 | 194,442 | 3,431,020 | 1,999,815 | 30,540,309 |
| maxSS | 0.526174 | SSP5-8.5_2021-2040 | 233,204 | 3,601,016 | 1,829,820 | 30,501,546 |
| 10th_percentile_presence | 0.568544 | SSP1-2.6_2021-2040 | 148,016 | 2,880,822 | 1,312,789 | 31,823,960 |
| 10th_percentile_presence | 0.568544 | SSP5-8.5_2021-2040 | 188,873 | 2,978,882 | 1,214,729 | 31,783,103 |
| equal_sensitivity_specificity | 0.572871 | SSP1-2.6_2021-2040 | 141,413 | 2,829,240 | 1,257,602 | 31,937,331 |
| equal_sensitivity_specificity | 0.572871 | SSP5-8.5_2021-2040 | 182,748 | 2,922,003 | 1,164,840 | 31,895,997 |

*Note. Gain, loss, stable suitable and stable unsuitable areas were calculated by comparing present-day and future binary suitability maps under the same threshold.*

**Supplementary Table S9d. Direction of suitable-area change across thresholds.**

| **Scenario** | **Number of thresholds** | **Number indicating contraction** | **Number indicating expansion** | **Percent-change range** | **Direction summary** |
| --- | --- | --- | --- | --- | --- |
| SSP1-2.6_2021-2040 | 3 | 3 | 0 | -66.19% to -60.07% | Contraction under all tested thresholds |
| SSP5-8.5_2021-2040 | 3 | 3 | 0 | -67.43% to -62.46% | Contraction under all tested thresholds |

*Note. Both future scenarios indicated contraction under all three thresholds tested.*
